# WHAT MAKES A NURSING HOME FEEL LIKE HOME? RESIDENT PERSPECTIVES

**DOI:** 10.1093/geroni/igz038.3380

**Published:** 2019-11-08

**Authors:** Diana Cater, Serena Hasworth, Diana White, Ozcan Tunalilar, Jaclyn Winfree

**Affiliations:** 1 Portland State University Institute on Aging, Portland, Oregon, United States; 2 Portland State University, Portland, Oregon, United States

## Abstract

Providing an environment that feels like home is increasingly a programmatic goal in nursing homes (NH), yet few NH studies have explored “home” as a concept through a large number of residents’ voices. In the current study, 294 residents living in 32 randomly selected NH in Oregon were asked if it felt like home. We followed up with “what makes it feel like home?” or “what would make it feel more like home?” Open-ended responses were classified via open coding. Six major themes emerged: relationships, meaningful possessions, quality of care, personal autonomy, physical environment, and the impossibility of it feeling like home. Overall, 26% percent of residents found that their current setting felt like home. Among residents who perceived their current setting as feeling like home, the most prominent theme was relationships with people at the nursing home (45% of responses). Residents also emphasized the importance of having family or other meaningful relationships from outside the nursing home (22% of all responses) for an environment that was home. Among those who responded that the nursing home did not feel like home, 32% reported that nothing could replace their home. Results indicate that NH can become home environments for their residents and provides additional evidence that relationship building, personal autonomy, and physical environment are likely critical pillars for creating and sustaining feelings of home. Future research will examine variations in responses described here, including resident or facility characteristics, and length of stay.

